# Melatonin Prevents NaAsO_2_-Induced Developmental Cardiotoxicity in Zebrafish through Regulating Oxidative Stress and Apoptosis

**DOI:** 10.3390/antiox11071301

**Published:** 2022-06-29

**Authors:** Rui Yan, Jie Ding, Yuanjie Wei, Qianlei Yang, Xiaoyun Zhang, Hairu Huang, Zhuoyue Shi, Yue Feng, Heran Li, Hengdong Zhang, Wenjun Ding, Yan An

**Affiliations:** 1 Jiangsu Key Laboratory of Preventive and Translational Medicine for Geriatric Diseases, Department of Toxicology, School of Public Health, Medical College of Soochow University, Suzhou 215123, China; 15895572995@163.com (R.Y.); dingjie0828@126.com (J.D.); wyj13340224565@163.com (Y.W.); qlyang@suda.edu.cn (Q.Y.); 15935916920@163.com (X.Z.); huanghairu217@163.com (H.H.); shizhuoyue1102@163.com (Z.S.); fengyue_2517@163.com (Y.F.); 2 Microwants International Ltd., Hong Kong, China; heranli@microwants.com; 3 Department of Occupational Disease Prevention, Jiangsu Provincial Center for Disease Control and Prevention, Jiangsu Preventive Medicine Association, Nanjing 210028, China; hdzhang220524@163.com; 4 Laboratory of Environment and Health, College of Life Sciences, University of Chinese Academy of Sciences, No. 19A Yuquan Road, Beijing 100049, China

**Keywords:** NaAsO_2_, melatonin, cardiac development, oxidative stress, apoptosis

## Abstract

Melatonin is an indoleamine hormone secreted by the pineal gland. It has antioxidation and anti-apoptosis effects and a clear protective effect against cardiovascular diseases. Our previous studies demonstrated that embryonic exposure to sodium arsenite (NaAsO_2_) can lead to an abnormal cardiac development. The aim of this study was to determine whether melatonin could protect against NaAsO_2_-induced generation of reactive oxygen species (ROS), oxidative stress, apoptosis, and abnormal cardiac development in a zebrafish (Danio rerio) model. We found that melatonin decreased NaAsO_2_-induced zebrafish embryonic heart malformations and abnormal heart rates at a melatonin concentration as low as 10^−9^ mol/L. The NaAsO_2_-induced oxidative stress was counteracted by melatonin supplementation. Melatonin blunted the NaAsO_2_-induced overproduction of ROS, the upregulation of oxidative stress-related genes (*sod2*, *cat*, *gpx*, *nrf2*, *ho-1*), and the production of antioxidant enzymes (Total SOD, SOD1, SOD2, CAT). Melatonin attenuated the NaAsO_2_-induced oxidative damage, DNA damage, and apoptosis, based on malonaldehyde and 8-OHdG levels and apoptosis-related gene expression (*caspase-3*, *bax*, *bcl-2*), respectively. Melatonin also maintained the control levels of heart development-related genes (*nkx2.5*, *sox9b*) affected by NaAsO_2_. In conclusion, melatonin protected against NaAsO_2_-induced heart malformations by inhibiting the oxidative stress and apoptosis in zebrafish.

## 1. Introduction

At present, nearly 200 million people worldwide are exposed to toxic and dangerous levels of arsenic through drinking water and diet [1]. The adverse effects of arsenic exposure on the cardiovascular system have become a global problem that seriously threatens the human public health [2]. Congenital heart disease (CHD) is a birth defect with a high incidence in China and it is one of the main causes of neonatal death [3]. The latest research shows that congenital heart disease is often caused by both genetic and environmental factors, and environmental factors have been considered of major importance [4,5]. Several clinical studies have shown that maternal arsenic exposure during pregnancy is closely related to the risk of congenital heart disease [6,7]. Animal experiments have also found that arsenic can cause an abnormal heart development in zebrafish embryos and fetal mice [8,9]. However, the specific molecular mechanism of arsenic-induced cardiac developmental toxicity is still unclear.

Melatonin (MT) is an indoleamine neuroendocrine hormone, secreted by the pineal gland, that has antioxidant and anti-apoptotic properties [10,11]. Population epidemiological data show that patients with heart-related diseases have low plasma levels of melatonin [12,13]. The physiological plasma melatonin concentration in newborns is very low, and exogenous administration of melatonin can alleviate several diseases in the neonatal period of humans, such as improving heart development [14,15]. Melatonin also has a significant protective effect for patients with cardiovascular diseases, especially against a variety of heart conditions induced by elevated oxidative stress levels [16]. Current research indicates a potential role for melatonin in both the treatment and prevention of congenital heart disease.

The zebrafish (Danio rerio) is an emerging vertebrate model. In this species, heart development begins at 5 h post-fertilization (hpf) in the late blastocyst stage and it is almost completed at 72 hpf [17]. In addition, early zebrafish embryos do not rely on the circulatory system and can survive severe heart malformations [18]. In this study, we aimed to investigate the protective effect of melatonin on NaAsO_2_-induced cardiac malformations using a zebrafish embryo model. The production of ROS, oxidative stress, DNA damage, and apoptosis were also studied to better understand the underlying molecular mechanisms of arsenic toxicity. These findings may help to further determine the potential of melatonin as an agent to prevent developmental cardiac diseases.

## 2. Materials and Methods

### 2.1. Fish Husbandry and Embryo Collection

An AB line wild-type zebrafish (Danio rerio) were purchased from the Zebrafish Resource Center in Wuhan, China and maintained under the following standard zebrafish culture system conditions: 28.5 °C, 14/10 light/dark cycle, pH 7.0–8.0. The water taken from the system was defined as “system water” and it was used for subsequent experiments. The zebrafish were fed three times daily. The zebrafish (male:female ratio was 1:1) were separated by sex and placed on either side of an isolation plate in spawning tanks. After the lights were turned on automatically the next day, the isolation plate was removed and the fish were allowed to mate freely. The embryos were collected 2 h later for subsequent experiments.

### 2.2. Chemical Exposure

The zebrafish embryos were randomly distributed into 6-well plates, with 30 embryos per well in 3 mL of system water, and exposed to 1 mmol/L (mM) NaAsO_2_ (Merck Drugs & Biotechnology, Darmstadt, Germany, purity 99.0%) in the absence or presence of melatonin (CAS 73-31-4, purity 99.0%, Sigma-Aldrich, St Louis, MO, USA) at different concentrations (10^−9^, 10^−8^, 10^−7^, 10^−6^ mol/L), with system water as the vehicle control [19,20]. The melatonin was added 3 h prior to the NaAsO_2_ for a total of 72 hpf. The system water, NaAsO_2_, and/or melatonin were renewed every 24 h during the exposure. Malformation and survival rates were recorded daily. Specifically, the heart malformations and heart rates were documented, and we chose an optimal melatonin concentration for subsequent studies based on the following two indicators: the concentration that could significantly prevent an increase in the cardiac malformation rates and a decrease in heart rates caused by sodium arsenite. The hearts were collected and dissected from the embryos at 72 hpf [21]. All procedures were approved by the Soochow University Animal Care and Use Committee, in accordance with the governmental regulations of China.

### 2.3. ROS Measurement

Dichlorodihydrofluorescein diacetate (DCFH-DA, Sigma-Aldrich) was used to measure the production of ROS in the heart of zebrafish embryos at 72 hpf. Approximately 20 zebrafish/groups were incubated with 20 μM DCFH-DA for 30 min in the dark at room temperature and then washed 3 times with system water. The images were taken with a fluorescence microscope (Olympus IX73, Olympus Ltd, Tokyo, Japan) and the fluorescence intensity of the heart area was analyzed using the ImageJ software.

### 2.4. Antioxidant Activity Assays

At 72 hpf, 40 larvae from each treatment group were collected and homogenized in 1% saline. After centrifugation (12,000× *g*), the supernatant was taken and the whole embryo catalase (CAT) and total SOD, SOD1, and SOD2 activities were measured using catalase activity and superoxide dismutase determination kits (Nanjing Jiancheng Institute of Bioengineering, Nanjing, China), according to the manufacturer’s instructions.

### 2.5. Acridine Orange (AO) Staining

After a 72 hpf exposure, acridine orange (AO, Sigma-Aldrich) was used to measure the level of cell apoptosis in the heart of zebrafish embryos. In the dark and at room temperature, approximately 20 zebrafish/groups were placed in 5 μg/mL AO for 30 min and then washed 3 times with water. A fluorescence microscope and ImageJ software were used to photograph the cardiac region and quantify the fluorescence intensity.

### 2.6. Oxidative Damage Assays

Following the preparation of the supernatant, outlined in Section 2.4, Malondialdehyde (MDA) activity was measured using a lipid peroxidation test kit (Beyotime Institute of Biotechnology, Haimen, China), following the manufacturer’s instructions. The 8-Hydroxydeoxyguanosine (8-OHdG) ELISA Kit (Elabscience Biotechnology Co., Ltd., Wuhan, China) was used to detect the level of 8-OHdG, a biomarker for oxidative DNA damage, according to the manufacturer’s protocol.

### 2.7. Quantitative Real-Time PCR (qPCR)

The total RNA was extracted from the isolated hearts (*n* = 100) using a Trizol reagent (TIANGEN, Beijing, China). The RNA quality and concentration were measured using a NanoDrop 2000 spectrophotometer (NanoDrop Technology, Wilmington, DE, USA). A total of 1000 ng of RNA was used for a cDNA synthesis reaction using a RevertAid First Strand cDNA Synthesis Kit (Thermo Scientific Fermentas, Waltham, MA, USA). Quantitative real-time PCR amplifications were carried out using an ABI 7500 q-PCR system (Applied Biosystems, Foster City, CA, USA) and a SYBR Green PCR Master Mix reagent kit (Roche, Shanghai, China). The transcription of *β-actin* was used as the internal control and the fold change from the control of the genes tested was calculated using the 2^−ΔΔCT^ method. The sequences of primers used in this study are presented in Table 1.

### 2.8. Statistical Analysis

All experiments were performed at least three times. The statistical methods used were one-way ANOVA followed by Dunnett’s or Turkey’s post-hoc test when appropriate. The results are expressed as mean ± SEM. The value *p* < 0.05 was used as the criterion for statistical significance.

## 3. Results

### 3.1. Melatonin Attenuated NaAsO_2_-Induced Heart Malformations

**Figure 1:**
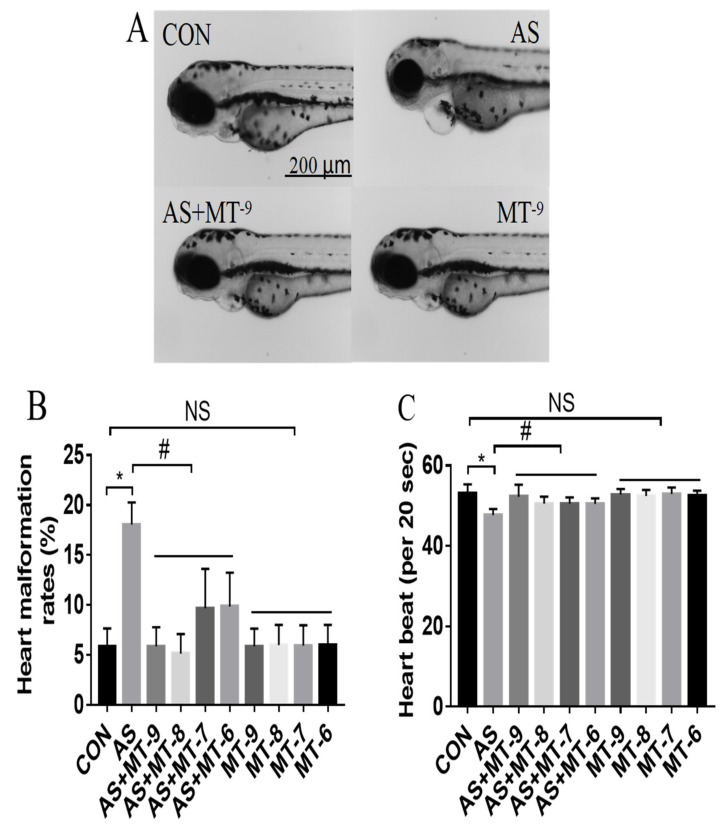
The effects of melatonin on the NaAsO_2_-induced heart malformations in zebrafish embryos at 72 hpf. (**A**) Images of zebrafish. (**B**) Heart malformation rate. (**C**) Heart rate. CON: system water as the vehicle control; AS: NaAsO_2_ at 1.0 mM; MT^−9^, MT^−8^, MT^−7^, MT^−6^: Melatonin at 10^−9^, 10^−8^, 10^−7^, 10^−6^ M; * *p* < 0.05, compared to CON; # *p* < 0.05, compared to MT; NS, compared to CON.

As shown in Figure 1A, we found that NaAsO_2_ at 1.0 mM without melatonin caused marked edema in the zebrafish heart, while melatonin alone at all concentrations produced no changes in the zebrafish heart. Melatonin with NaAsO_2_ decreased the pericardial edema caused by the NaAsO_2_. Compared with the control group, NaAsO_2_ caused a 3-fold increase in the incidence of cardiac malformations and a significant decrease in the heart rates by approximately 18 beats/min. The different concentrations of melatonin alone had no effect on the heart rates in zebrafish. The data showed that after the pretreatment with the different concentrations, melatonin effectively prevented the cardiac malformations and abnormal heart rates caused by the NaAsO_2_ and the preventive effect is consistent between the different concentrations of melatonin. Therefore, the lowest melatonin concentration of 10^−9^ mol/L was used for all subsequent experiments (Figure 1B,C).

### 3.2. Melatonin Inhibited NaAsO_2_-Induced Oxidative Stress

**Figure 2:**
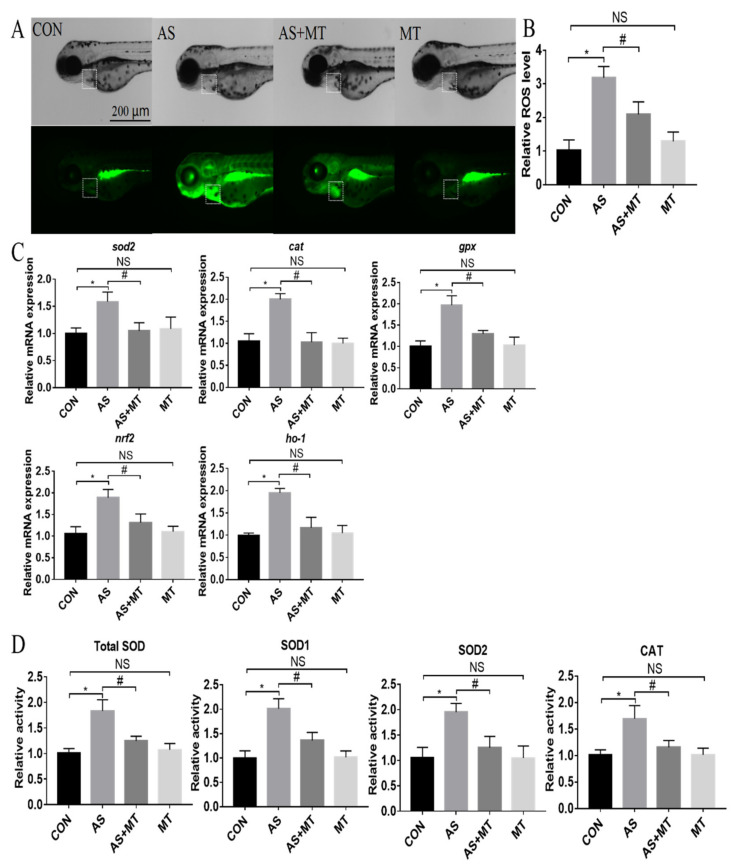
The effects of melatonin on the NaAsO_2_-induced oxidative stress changes in zebrafish embryos at 72 hpf. (**A**) ROS signals in the heart area and heart regions are represented by the dotted squares. (**B**) Quantification of ROS signals in heart area. (**C**) Relative expression levels of oxidative stress-related genes. (**D**) Relative enzyme activities. CON: system water as the vehicle control; AS: NaAsO_2_ at 1.0 mM; MT^−9^: Melatonin at 10^−9^ M. * *p* < 0.05, compared to CON; # *p* < 0.05, compared to MT; NS, compared to CON.

Compared with the control group, NaAsO_2_ caused a significant nearly 3-fold increase in ROS production, while melatonin pretreatment blunted but did not completely prevent the increase in ROS (Figure 2A,B). Furthermore, NaAsO_2_ exposure significantly upregulated the mRNA expression of oxidative stress-related genes (*sod2*, *cat*, *gpx*, *nrf2*, *ho-1*) and these genes remained at control levels with the melatonin pretreatment (Figure 2C). Predictably, the oxidative stress-related enzyme activities of the total SOD, SOD1, SOD2, and CAT were increased following the exposure to NaAsO_2_ but remained at control levels with the melatonin pretreatment (Figure 2D).

### 3.3. Melatonin Prevented NaAsO_2_-Induced Oxidative Damage and Apoptosis

**Figure 3:**
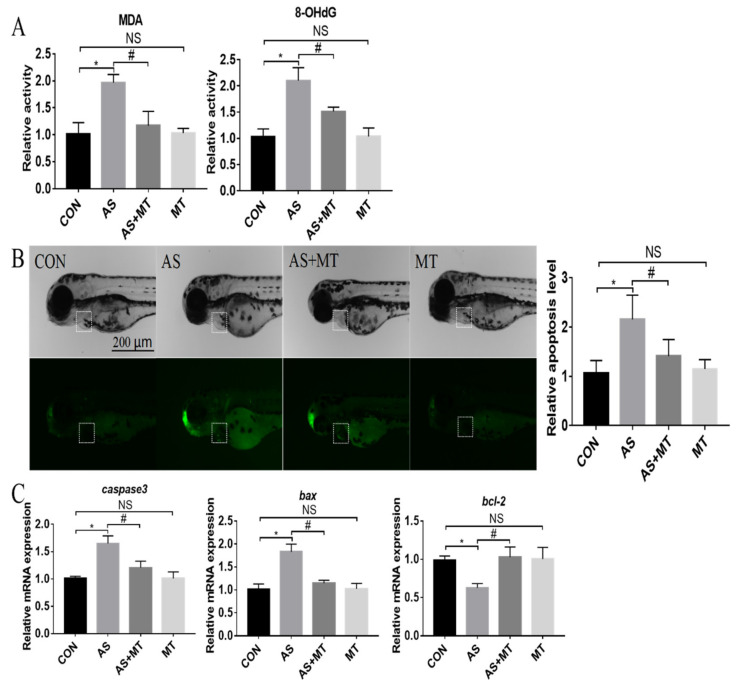
The effects of melatonin on the NaAsO_2_-induced oxidative damage and apoptosis changes in zebrafish embryos at 72 hpf. (**A**) Oxidative damage in zebrafish embryos. (**B**) Representative images stained by AO and the quantification of apoptosis signals in heart area. Heart regions are represented by the dotted squares. (**C**) Relative expression levels of apoptosis-related genes. CON: system water as the vehicle control; AS: NaAsO_2_ at 1.0 mM; MT^−9^: Melatonin at 10^−9^ M. * *p* < 0.05, compared to CON; # *p* < 0.05, compared to MT; NS, compared to CON.

As shown in Figure 3A, when using MDA and 8-OHdG as oxidative lipid- and DNA-damaging indicators, respectively, NaAsO_2_ induced significant changes in the MDA and 8-OHdG relative activity compared with the control group, while melatonin inhibited these changes caused by the NaAsO_2_. Based on AO staining, NaAsO_2_ caused a 2-fold increase in the level of apoptosis in the heart region of zebrafish embryos, which was partially blocked by the melatonin pretreatment (Figure 3B). Furthermore, NaAsO_2_ significantly upregulated the mRNA expression of apoptosis-related genes (*caspase3*, *bax*) and downregulated the mRNA expression of the apoptosis-related gene bcl-2. These genes remained at control levels with the melatonin pretreatment (Figure 3C).

### 3.4. Melatonin Prevented NaAsO_2_-Induced Effects in Heart Development-Related Genes

**Figure 4:**
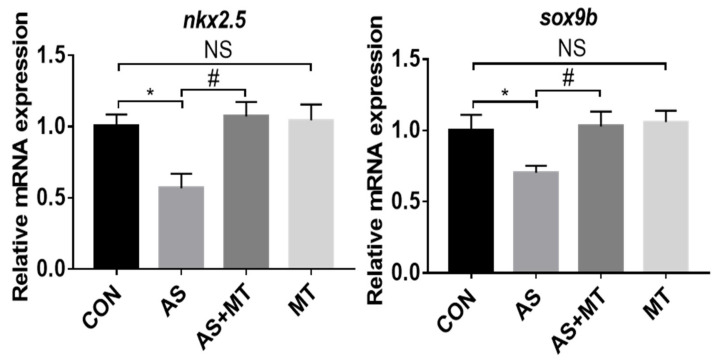
The effects of melatonin on the NaAsO_2_-induced relative mRNA expression levels of the genes essential for heart development in zebrafish embryos at 72 hpf. CON: system water as the vehicle control; AS: NaAsO_2_ at 1.0 mM; MT^−9^: Melatonin at 10^−9^ M. * *p* < 0.05, compared to CON; # *p* < 0.05, compared to MT; NS, compared to CON.

The expression of heart development-related genes (*nkx2.5*, *sox9b*) all showed that NaAsO_2_ exposure led to significant decreases in the gene expression levels, while the melatonin pretreatment maintained these related genes at control expression levels (Figure 4).

## 4. Discussion

Increasing evidence has indicated that arsenite has the potential to induce various cardiac diseases, especially congenital heart disease caused by arsenite exposure in the periconception period, which seriously endangers the health of infants and young children, but the underlying mechanisms are still unclear [22]. Current studies are commonly done using mouse models, although a few studies have been done using zebrafish. Our previous studies using zebrafish showed that arsenite lead to abnormal cardiac development by interfering with the GH/IGF axis, which is manifested by the upregulation of GH hormone levels and the significant downregulation of the related genes and their receptors and transporters [23]. Melatonin has cardioprotective effects, including prevention of alcohol- and nicotine-induced heart toxicity [24,25], and alleviation of cardiac ischemia/reperfusion (I/R)-induced oxidative damage [26]. At present, melatonin is a preventive agent in different species and cell lines from various environmental compounds within a concentration range between 10^−12^–10^−3^ mol/L and its application concentration is 10^−12^–10^−6^ mol/L in cells, 10^−6^–10^−3^ mol/L in mice, and 10^−9^–10^−6^ mol/L in zebrafish [27,28,29,30]. In our study, the concentration of 10^−9^–10^−6^ mol/L was used to investigate whether melatonin could play a preventive role in arsenite-induced cardiac injury. The results showed that melatonin pretreatment concentration of 10^−9^–10^−6^ mol/L was effective at counteracting arsenite-induced cardiac malformation and maintained cardiac malformation and heart rates at control levels. In order to further explore the possible mechanism of melatonin preventing arsenite-induced cardiac injury, we chose the lowest melatonin concentration of 10^−9^ mol/L for subsequent experiments.

There is extensive literature on arsenite-induced ROS generation, and oxidative stress is a common mechanism of arsenite-induced damage [31]. During the early stages of life, organisms are sensitive to oxidative stress caused by ROS and a recent study showed that even background environmental concentrations of arsenite could cause oxidative damage [32]. Melatonin has the effects of scavenging free radicals, antioxidation, and anti-apoptosis [33,34]. A recent study suggested that melatonin effectively prevented PM2.5-induced cardiac dysfunction and fibrosis via inhibiting mitochondrial oxidative injury and regulating the SIRT3-mediated SOD2 deacetylation [28]. Our experiments confirmed that arsenite induces oxidative stress, including overproduction of ROS, and changes in the expression of antioxidant genes (*sod2*, *cat*, *gpx*, *nrf2*, *ho-1*) and antioxidant enzymes (total SOD, SOD1, SOD2, and CAT) related to the antioxidant defense system in response to ROS. Our results further demonstrated that pretreatment with melatonin prevented these changes induced by arsenite with its antioxidant properties. Arsenite induced the overproduction of ROS that activated the overall antioxidant defense system in zebrafish, resulting in increased levels of activity and mRNA expression of antioxidant enzymes. Whereas melatonin pretreatment prevented the overproduction of ROS and restored the oxidative-antioxidant system to normal levels in zebrafish. Similar mechanisms for the effects on the organisms of other environmental compounds have been reported in recent studies [19,35]. However, oxygen free radicals play an indispensable role in the normal physiological processes of the body, and non-selective quenching of all free radicals in the cells may be harmful, but selectively scavenging or reducing the mitochondrial ROS production could be beneficial for the prevention and treatment of ROS-related diseases [36,37]. Various studies have shown that SIRT3, the most important deacetylase in mitochondria, is an effective intervention target for the prevention and treatment of oxidative stress-related diseases [38,39]. In addition, studies have also shown that SIRT3 is a potential intervention target for a variety of cardiovascular diseases caused by myocardial ischemia-reperfusion injuries, chemotherapy drugs, and environmental toxicants and it is currently a focal molecule in the field of cardiovascular protection research [11,40]. Therefore, in future research, we will focus on whether SIRT3 mediates the preventive effect of melatonin on arsenite-induced developmental cardiotoxicity in zebrafish.

Excessive production of ROS resulting in oxidative damage at the cellular level is inextricably linked to a variety of adverse outcomes, such as cardiovascular disease [41,42]. Herein we examined changes in the MDA and 8-OHdG levels across groups as indices of the lipid-damaging and DNA-damaging effects of arsenite and found that melatonin blocked these changes. Studies have shown that DNA damage is a trigger for apoptosis and it is believed that the synergistic effect of DNA damage and apoptotic cell death is conducive to tissue remodeling in vertebrate hearts [43]. In this study, we showed that the level of apoptosis in zebrafish embryos exposed to arsenite was increased. This was demonstrated by increased AO staining, an increase in the apoptotic terminal cleavage enzyme *caspase3*, an increase in the pro-apoptotic gene *bax*, and a decrease in the anti-apoptotic gene *bcl-2*. Pretreatment with melatonin attenuated all indices of arsenite-induced apoptosis.

The *nkx2.5* and *sox9b* genes play important regulatory roles in the differentiation of cardiac precursor cells, the circularization of the heart, the formation of the atrioventricular septum, and the maintenance of mature cardiac function [44,45]. We examined the mRNA levels of these genes and found that melatonin blunted the arsenite-induced low expression.

## 5. Conclusions

**Figure 5:**
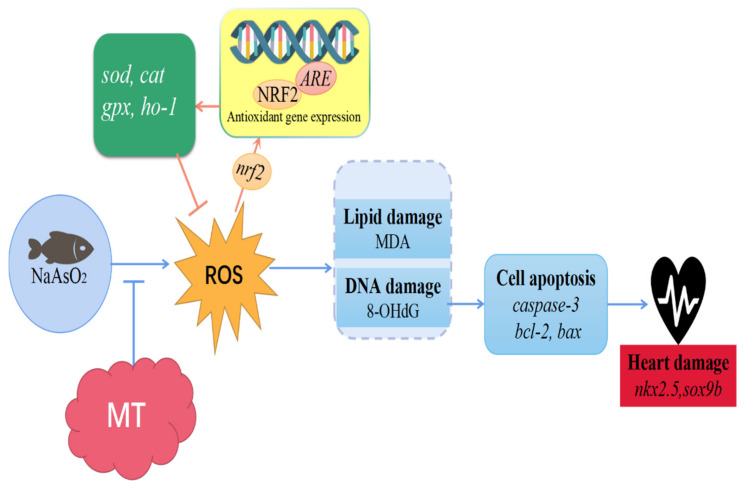
A model indicating that the protection mediated by melatonin reduced the developmental cardiotoxicity of zebrafish embryos induced by NaAsO_2_. Melatonin inhibited the overproduction of ROS caused by the NaAsO_2_ exposure, restored the antioxidant defense system, reduced oxidative damage and cell apoptosis, thereby preventing the developmental cardiotoxicity of zebrafish.

Using the zebrafish embryo model, we demonstrated that melatonin prevented arsenite-induced cardiac defects. Moreover, arsenite-induced oxidative stress, oxidative damage, and apoptosis were counteracted by melatonin. (Figure 5). The findings from this study will increase our knowledge regarding the protective effect of melatonin in preventing arsenite-induced cardiac developmental disorders.

## Figures and Tables

**Table 1:** Sequences of primers for the genes tested.

Gene Name		Sequence of the Primer (5′–3′)
*β-actin*	NM_131031.2	Forward: CGAGCAGGAGATGGGAACC
		Reverse: CAACGGAAACGCTCATTGC
*bax*	NM_131562.2	Forward: GGCTATTTCAACCAGGGTTCC
		Reverse: TGCGAATCACCAATGCTGT
*bcl2*	NM_001030253.2	Forward: AGGAAAATGGAGGTTGGGATG
		Reverse: TGTTAGGTATGAAAACGGGTGGA
*caspase3*	NM_131877.3	Forward: CCGCTGCCCATCACTA
		Reverse: ATCCTTTCACGACCATCT
*cat*	NM_130912.2	Forward: AGGGCAACTGGGATCTTACA
		Reverse: TTTATGGGACCAGACCTTGG
*gpx*	NM_001007281.2	Forward: AGATGTCATTCCTGCACACG
		Reverse: AAGGAGAAGCTTCCTCAGCC
*ho-1*	NM_001127516.1	Forward: GGAAGAGCTGGACAGAAACG
		Reverse: CGAAGAAGTGCTCCAAGTCC
*nkx2.5*	NM_131421.2	Forward: GCATCAGAGCTTGGTGAACA
		Reverse: ATGCGCACGCATAAACATTA
*nrf2*	NM_182889.1	Forward: GACAAAATCGGCGACAAAAT
		Reverse: TTAGGCCATGTCCACACGTA
*sod2*	NM_199976.1	Forward: GCTTGGGATAGATGTCTGGG
		Reverse: CTTGGAAACGCTCGCTGA
*sox9b*	NM_131644.1	Forward: CGAGAAGCGTCCGTTTGTG
		Reverse: CCGTCTGGGCTGGTATTTGTA

## Data Availability

Data is contained within the article.
